# Development and validation of a nomogram to predict overall survival in patients with incidental gallbladder cancer: A retrospective cohort study

**DOI:** 10.3389/fonc.2022.1007374

**Published:** 2023-01-24

**Authors:** Zhi-Hua Xie, Xuebing Shi, Ming-Qi Liu, Jinghan Wang, Yong Yu, Ji-Xiang Zhang, Kai-Jian Chu, Wei Li, Rui-Liang Ge, Qing-Bao Cheng, Xiao-Qing Jiang

**Affiliations:** ^1^ Department I of Biliary Tract Surgery, Eastern Hepatobiliary Surgery Hospital, Naval Medical University, Shanghai, China; ^2^ Department of Hepatopancreatobiliary Surgery, East Hospital, Tongji University, Shanghai, China

**Keywords:** gallbladder cancer, re-resection, nomogram, overall survival, incidental gallbladder cancer

## Abstract

**Objective:**

The aim of this study was to develop and validate a nomogram to predict the overall survival of incidental gallbladder cancer.

**Methods:**

A total of 383 eligible patients with incidental gallbladder cancer diagnosed in Shanghai Eastern Hepatobiliary Surgery Hospital from 2011 to 2021 were retrospectively included. They were randomly divided into a training cohort (70%) and a validation cohort (30%). Univariate and multivariate analyses and the Akaike information criterion were used to identify variables independently associated with overall survival. A Cox proportional hazards model was used to construct the nomogram. The C-index, area under time-dependent receiver operating characteristic curves and calibration curves were used to evaluate the discrimination and calibration of the nomogram.

**Results:**

T stage, N metastasis, peritoneal metastasis, reresection and histology were independent prognostic factors for overall survival. Based on these predictors, a nomogram was successfully established. The C-index of the nomogram in the training cohort and validation cohort was 0.76 and 0.814, respectively. The AUCs of the nomogram in the training cohort were 0.8, 0.819 and 0.815 for predicting OS at 1, 3 and 5 years, respectively, while the AUCs of the nomogram in the validation cohort were 0.846, 0.845 and 0.902 for predicting OS at 1, 3 and 5 years, respectively. Compared with the 8th AJCC staging system, the AUCs of the nomogram in the present study showed a better discriminative ability. Calibration curves for the training and validation cohorts showed excellent agreement between the predicted and observed outcomes at 1, 3 and 5 years.

**Conclusions:**

The nomogram in this study showed excellent discrimination and calibration in predicting overall survival in patients with incidental gallbladder cancer. It is useful for physicians to obtain accurate long-term survival information and to help them make optimal treatment and follow-up decisions.

## 1 Introduction

Gallbladder cancer (GBC) is a rare malignancy with a documented incidence of 1.13 per 100,000 (1). Most patients are diagnosed with advanced incurable disease with a poor prognosis. The 5-year overall survival (OS) for stage III was 22.1% to 25.7% and 6.7% to 15.7% for stage IV patients (2). Radical resection is the only potential cure for GBC patients, especially those in early stages, who are most frequently diagnosed incidentally. In particular, with the widespread adoption of laparoscopic cholecystectomy, the number of incidentally gallbladder cancers (IGBCs) discovered after cholecystectomy for presumed benign disease has increased dramatically, accounting for 1.6% of all cholecystectomies (3). Due to the predisposition of port-site metastasis, peritoneal metastasis and the possibility of tumor residual in the liver bed and/or regional lymph nodes after initial cholecystectomy, the optimal management of IGBCs after the index cholecystectomy is a challenge which has attracted physicians’ attention.

Although the extent and timing of reresection for IGBC remain controversial, reoperation has been recommended because of improved survival in retrospective studies. The rationale behind reresection is not only to remove any residual disease but also to restage the disease accurately, which may be instrumental in achieving tumor-free margins, guiding adjuvant therapy and predicting prognosis (4–6). However, the existing survival prediction models of GBC do not take its specific characteristics (such as reresection and time to reoperation) into account due to its low incidence (7–13), and may not be able to provide accurate survival predictions for patients with IGBC and reduce the prognostic value of the 8th edition of the American Joint Committee on Cancer (AJCC) staging system model. Therefore, in this special group of patients, it is necessary to identify independent prognostic factors associated with IGBC survival and develop an appropriate model to accurately predict the survival rate of IGBC.

Recently, user-friendly and intuitive nomograms that can accurately predict overall survival have been widely used to evaluate the prognosis of various cancers. In this study, univariate and multivariate analyses were used to explore the independent prognostic factors of IGBC based on the clinicopathological data collected from our center in the past decade. Next, a nomogram was established to predict OS, and the accuracy and precision of the nomogram in the training and validation sets were evaluated by receiver operating characteristic (ROC) curves and calibration curves, respectively.

## 2 Materials and methods

### 2.1 Patient selection

This study was approved by the institutional Review Board of our Ethics Committee, and informed patient consent was obtained (No. EHBHKY2022-K-025). The patients who underwent index cholecystectomy and were diagnosed with IGBC in Eastern Hepatobiliary Surgery Hospital from 2011 to 2021 and those who were first diagnosed with IGBC in other hospitals and underwent reresection for curable purposes in our hospital during the period were retrospectively analyzed. All enrolled cases were randomly divided into two datasets: 70% of eligible cases were allocated to the training cohort (n=269), and 30% were allocated to the validation cohort (n=114). The inclusion criteria for both cohorts were all patients diagnosed with incidental gallbladder cancer, defined as patients with no preoperative suspicion of GBC but pathologically confirmed gallbladder malignant tumor after cholecystectomy. Patients who were under the age of 18 years at diagnosis or lacked follow-up information were excluded. Clinical information such as sex, age at diagnosis, histology type, T stage, N metastasis, peritoneal metastasis, etc., were reviewed from medical records. The cutoff value of the time to reoperation was defined as the median time (19 days). The histological classification was adenocarcinoma or nonadenocarcinoma (adenosquamous or squamous). N metastasis was described as either negative or positive lymph node status. M metastasis was described as either negative or positive distant metastasis. Resection margin R was described as either negative (R0) or positive (R1/R2). Overall survival was chosen as the endpoint of interest, with dates calculated from the time of first surgery to death from any cause or the last follow-up on January 1, 2022.

### 2.2 Statistical analysis

Descriptive statistics were used to summarize all clinical features. χ^2^ or Fisher’s exact tests were performed to assess the distribution of basic categorical variables of patients in the training and validation cohorts, as appropriate. Potential prognostic variables with p values <0.1 identified in univariable Cox analyses were further selected and included in multivariable Cox regression analyses. Stepwise backward model selection was performed based on Akaike information criterion (AIC) values. Variables with two-sided p values <0.05 were considered as statistically significant and were identified as independent prognostic factors to construct a nomogram of the prediction model. In the training and validation cohorts, the nomogram was validated both internally and externally with 500-bootstrap resampling.

Discrimination and calibration were used to evaluate the predicted OS performance of the nomogram. Harrell’s concordance index (C-index) was calculated to measure the difference between the observed outcomes and the nomogram predictions on a scale of 0.5 to 1.0, where 0.5 indicated no discrimination at all and 1.0 indicated a perfect fit. Calibration curves were visualized to compare the predicted and observed probabilities of OS at 1, 3 and 5 years. Furthermore, time-dependent ROC curves were generated to compare the power of the nomogram model with the 8th edition of the AJCC TNM staging system model. Missing data were completed with multiple imputation using the ‘mice’ package with default values. Statistical analyses were performed using version R 4.1.1 (R Foundation for Statistical Computing, Vienna, Austria). The related R packages ‘rms’, ‘foreign’, ‘VIM’, ‘epiDisplay’, ‘dplyr’, ‘mice’, ‘survival’, ‘survivalROC’, ‘forestplot’, and ‘caret’ were applied to create and evaluate the nomogram. This study was designed according to the Transparent Reporting of a multivariable prediction model for Individual Prognosis or Diagnosis (TRIPOD) guidelines.

## 3 Results

### 3.1 Clinical characteristics

A total of 383 patients with incidental gallbladder cancer who met the inclusion criteria were identified from 2011 to 2021. They were randomly divided into the training cohort (n=269, 70%) and validation cohort (n=114, 30%). All patients underwent surgical resection, of whom approximately 84.1% underwent reresection. The median follow-up was 31.3 months, with a range of 1.3 to 136 months. Detailed baseline characteristics of patients in each cohort are shown in **Table 1**. Approximately 64% of the cases were female, and 31.6% were male. There were slightly more patients under the age of 60 than those over the age of 60 (51.4% *vs*. 48.6%). Most of the patients had T2-T3 stages (315 cases, 82.2%) and adenocarcinomas (340 cases, 88.8%). More importantly, in these 383 patients, upon reresection, 9.7% developed distant metastases, 19.3% developed lymph node metastases, and 6.3% developed peritoneal metastases. Among the 383 cases, 141 (36.8%) had chronic disease. In addition, the median time to reoperation was 19 [InterQuartile Range (IQR), 12-26.5] days, and a total of 182 (47.5%) patients underwent reoperation within 19 days from their initial cholecystectomy.

**Table 1:** Characteristics of incidental gallbladder cancer patients in the Training and Validation set.

Characteristics	Training set	Validation set	Total	statistic	*P*
	269 (%)	114 (%)	383 (%)		
**sex**				χ2= 1.4	0.237
female	167 (62.1)	78 (68.4)	245 (64)		
male	102 (37.9)	36 (31.6)	138 (36)		
**age60**				χ2 = 0.09	0.761
<60year	137 (50.9)	60 (52.6)	197 (51.4)		
>=60year	132 (49.1)	54 (47.4)	186 (48.6)		
**T stage**				χ2 = 1.77	0.777
T1	24 (8.9)	8 (7)	32 (8.4)		
T2	103 (38.3)	50 (43.9)	153 (39.9)		
T3	118 (43.9)	44 (38.6)	162 (42.3)		
T4	11 (4.1)	5 (4.4)	16 (4.2)		
NA	13 (4.8)	7 (6.1)	20 (5.2)		
**M metastasis**				χ2 = 1.28	0.528
No	234 (87)	95 (83.3)	329 (85.9)		
Yes	23 (8.6)	14 (12.3)	37 (9.7)		
NA	12 (4.5)	5 (4.4)	17 (4.4)		
**N metastasis**				χ2 = 1.94	0.379
No	209 (77.7)	81 (71.1)	290 (75.7)		
Yes	48 (17.8)	26 (22.8)	74 (19.3)		
NA	12 (4.5)	7 (6.1)	19 (5)		
**TNM stage**				χ2= 2.12	0.713
I	23 (8.6)	8 (7)	31 (8.1)		
II	88 (32.7)	38 (33.3)	126 (32.9)		
III	114 (42.4)	43 (37.7)	157 (41)		
IV	34 (12.6)	20 (17.5)	54 (14.1)		
NA	10 (3.7)	5 (4.4)	15 (3.9)		
**Peritoneal metastasis**				Fisher's	0.544
No	248 (92.2)	108 (94.7)	356 (93)		
Yes	19 (7.1)	5 (4.4)	24 (6.3)		
NA	2 (0.7)	1 (0.9)	3 (0.8)		
**Lymphatic invasion**				χ2= 1.26	0.533
No	247 (91.8)	101 (88.6)	348 (90.9)		
Yes	10 (3.7)	7 (6.1)	17 (4.4)		
NA	12 (4.5)	6 (5.3)	18 (4.7)		
**Perineural invasion**				χ2= 0.44	0.802
No	228 (84.8)	98 (86)	326 (85.1)		
Yes	29 (10.8)	10 (8.8)	39 (10.2)		
NA	12 (4.5)	6 (5.3)	18 (4.7)		
**Vascular invasion**				χ2= 0.98	0.612
No	250 (92.9)	103 (90.4)	353 (92.2)		
Yes	7 (2.6)	5 (4.4)	12 (3.1)		
NA	12 (4.5)	6 (5.3)	18 (4.7)		
**Resection margin R**				Fisher's	0.399
negative	205 (76.2)	87 (76.3)	292 (76.2)		
positive	64 (23.8)	26 (22.8)	90 (23.5)		
NA	0 (0)	1 (0.9)	1 (0.3)		
**Chronic disease**				χ2 = 0.21	0.648
No	168 (62.5)	74 (64.9)	242 (63.2)		
Yes	101 (37.5)	40 (35.1)	141 (36.8)		
**Time to reoperation**				χ2= 0.03	0.853
<19d	127 (47.2)	55 (48.2)	182 (47.5)		
>=19d	142 (52.8)	59 (51.8)	201 (52.5)		
**Re-resection**				χ2 = 0.07	0.797
No	42 (15.6)	19 (16.7)	61 (15.9)		
Yes	227 (84.4)	95 (83.3)	322 (84.1)		
**Histology**				χ2= 1.3	0.523
Ade	242 (90)	98 (86)	340 (88.8)		
Nonade	8 (3)	5 (4.4)	13 (3.4)		
NA	19 (7.1)	11 (9.6)	30 (7.8)		

NA, not available; N metastasis, lymph node metastasis; M metastasis, distant metastasis; Ade, adenocarcinoma; Nonade, Noadenocarcinoma.

### 3.2 Identification of prognostic factors

**Figure 1:**
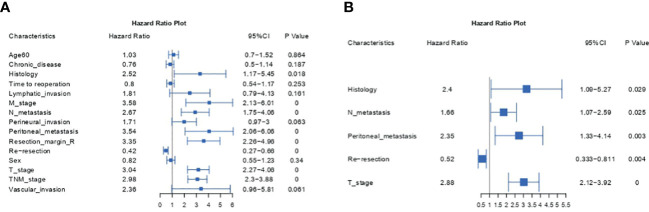
Forest plots of the univariate **(A)** and multivariate **(B)** Cox analyses.

To identify prognostic factors associated with OS before constructing a nomogram model, we employed univariate and multivariate Cox regression analyses. **Table 2** and **Figure 1** show the detailed results of the univariate and multivariate analyses in the training cohort. Univariate analysis found that histology, M metastasis, N metastasis, perineural invasion, peritoneal metastasis, resection margin R, reresection, T stage, TNM stage and vascular invasion were associated with OS. Variables with *P value*s *<*0.1 were considered as statistically significant. Subsequently, these ten meaningful variables were put into a multivariate Cox regression model using a backward stepwise method. Based on multivariate analysis, five variables (histology, N metastasis, peritoneal metastasis, reresection and T stage) were finally considered as independent prognostic factors with a *P* value <0.05 and a minimum AIC value of 1001.06.

**Table 2:** Univariable and multivariable Cox analyses of OS in patients with IGBC.

Variable	Univariate analysis				Multivariate analysis		
	HR	95% CI	P	HR	95% CI	β coef	P
Age60 (>=60year vs <60year)	1.03	0.7-1.52	0.864				
Chronic disease (Yes vs No)	0.76	0.5-1.14	0.187				
Histology (Nonade vs Ade)	2.52	1.17-5.45	0.018	2.3989	1.0919-5.2701	0.8750	0.02933 *
Time to reoperation (>1=19 vs <19)	0.8	0.54-1.17	0.253				
Lymphatic invasion (Yes vs No)	1.81	0.79-4.13	0.161				
M stage (Yes vs No)	3.58	2.13-6.01	0				
N metastasis (Yes vs No)	2.67	1.75-4.06	0	1.6627	1.0667-2.5919	0.5085	0.02477 *
Perineural invasion (Yes vs No)	1.71	0.97-3	0.063				
Peritoneal metastasis (Yes vs No)	3.54	2.06-6.06	0	2.3475	1.3308-4.1411	0.8534	0.00321 **
Resection margin R (Yes vs No)	3.35	2.26-4.96	0				
Re-resection (Yes vs No)	0.42	0.27-0.66	0	0.5195	0.3327-0.8112	-0.6549	0.00397 **
Sex (Male vs Female)	0.82	0.55-1.23	0.34				
T stage (IV vs III vs II vs I)	3.04	2.27-4.06	0	2.8838	2.1210-3.9209	1.0591	1.41e-11 ***
TNM stage (IV vs III vs II vs I)	2.98	2.3-3.88	0				
Vascular invasion (Yes vs No)	2.36	0.96-5.81	0.061				

OS, overall survival; IGBC, incidentally gallbladder cancer; Ade, adenocarcinoma; Nonade, Noadenocarcinoma.

### 3.3 Construction of the prognostic nomogram

**Figure 2:**
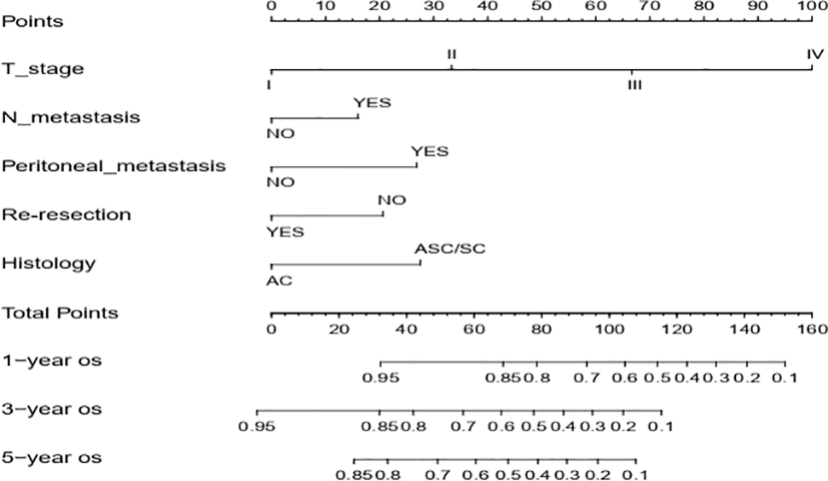
Nomogram for estimating the 1-, 3-, 5-year OS of IGBC patients. N metastasis, lymph node metastasis; AC, adenocarcinoma; ASC/SC, adenosquamous or squamous; OS, overall survival.

**Figure 3:**
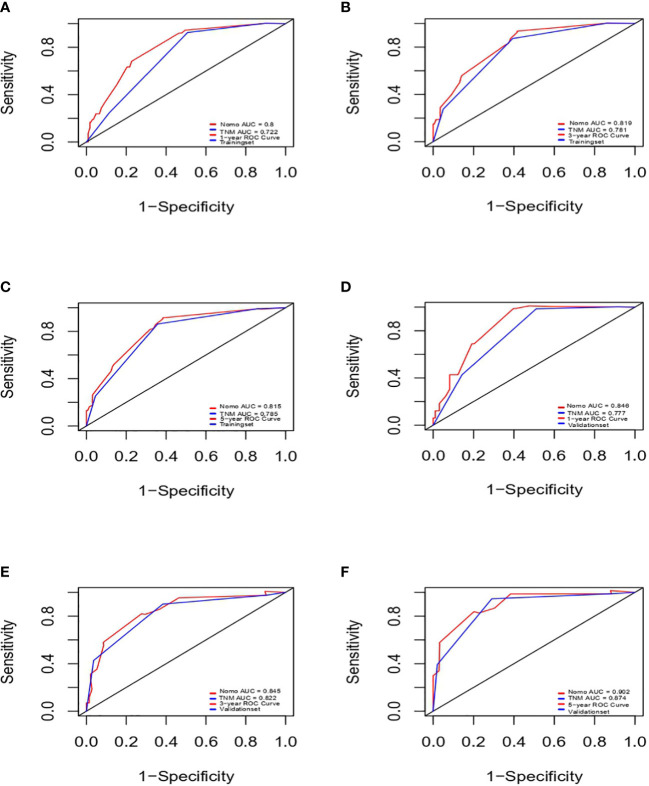
ROCs of IGBC for predicting OS at 1-, 3-, 5-year in the training **(A–C)** and validation set **(D–F)**, respectively. ROC, receiver operating characteristic; AUC, area under the curve; IGBC, incidentally gallbladder cancer; OS, overall survival.

**Figure 4:**
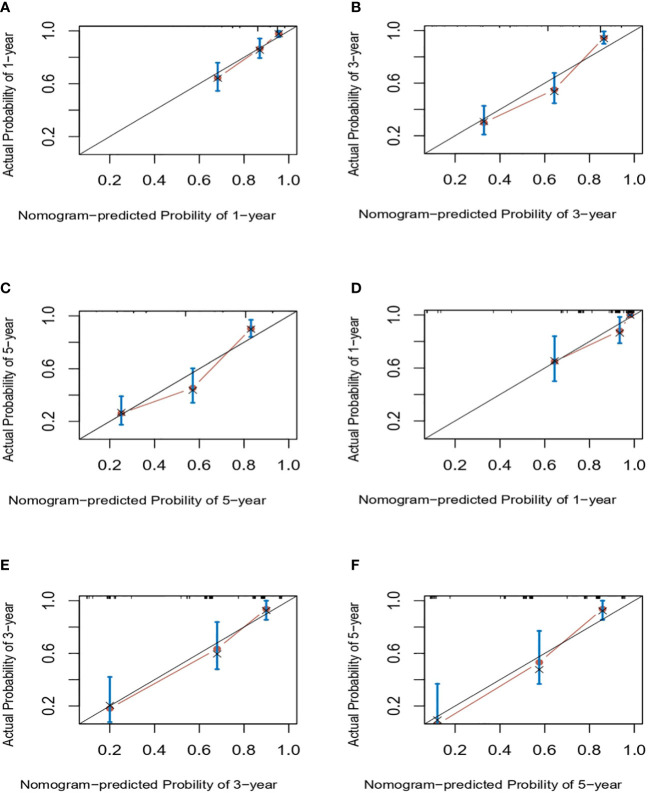
Calibration curves for predicting IGBC OS at 1-, 3-, 5-year in the training **(A–C)** and validation set **(C–E)**, respectively. IGBC, incidentally gallbladder cancer; OS, overall survival.

Next, we successfully developed a nomogram model to predict OS at 1, 3, and 5 years based on the above five identified independent variables, as shown in **Figure 2**. According to the total subscale at the bottom, the probabilities of 1-, 3-, and 5-year OS were simply calculated from the sum of the scores for each individual variable. Harrell’s C-index, time-dependent ROC curves (**Figure 3**) and calibration curves (**Figure 4**) were used to evaluate the established nomogram model. The C-index value of the nomogram was 0.76 [95% confidence interval (CI), 0.72-0.80] in the training cohort and 0.814 (95% CI, 0.76-0.87) in the validation cohort. Time-dependent ROC curves were used to compare the sensitivity and specificity between the predictive model and the TNM staging model. The areas under the curve (AUCs) of the nomogram for predicting OS at 1, 3, and 5 years were 0.8, 0.819 and 0.815 in the training cohort and 0.846, 0.845 and 0.902 in the validation cohort, respectively. Meanwhile, the 1-, 3- and 5-year AUC values of the TNM staging model were 0.722, 0.781 and 0.785 in the training cohort and 0.777, 0.822 and 0.874 in the validation cohort, respectively. As shown in **Figure 3**, in the training and validation cohorts of 1-, 3- and 5-year OS, the nomogram model showed better discriminative power and larger AUCs than the TNM staging model, illustrating that the nomogram model exhibited a more powerful discrimination. Meanwhile, calibration curves illustrating the relationship between predicted and actual OS probabilities were tested with 500 bootstrap resamples in both the training and validation cohorts. Calibration plots showed that OS prediction at 1, 3, and 5 years for both cohorts was in excellent agreement with actual observations. Taken together, the nomogram model demonstrated good discriminative and calibration power for predicting 1-, 3-, and 5-year OS in IGBC.

## 4 Discussion

Gallbladder cancer is an aggressive disease with a dismal prognosis. It is usually occult onset with an asymptomatic course that is not easily discovered in the early stages before operation. Most incidental gallbladder cancers were occasionally diagnosed after cholecystectomy, and a few were discovered during surgery. In recent decades, with the rapid increase in the number of patients undergoing laparoscopic cholecystectomy, the gradual increase in the incidence of IGBC is of concern. However, existing models and the latest AJCC TNM staging system for predicting survival in GBC that do not specifically consider IGBC may not be applicable to IGBC (7–14). Due to its user-friendly graphical interface and the integration of multiple easily accessible variables, the nomogram has been increasingly popular and widely used for personalized cancer prediction of various cancers. In this study, we first developed a nomogram model to predict survival for IGBC. Based on univariate and multivariate analyses, we identified five factors (T stage, N metastasis, peritoneal metastasis, reresection, histology) that were independently associated with overall survival.

T stage in our study was one of the top five independent prognostic factors that has also been identified in previous studies of IGBC (6, 15–17). Residual disease was considered as one of the most important characteristics of IGBC; in statistics, approximately 35% ~ 50.8% of patients had residual disease (RD) (4, 18), and T stage was closely associated with residual disease and proved to be an excellent predictor of residual disease in IGBC^18^. It was reported that approximately 20% of T1b, 23.8% of T2, and 71.7% of T3 of IGBC patients had accompanying RD (19). Evidently, R0 resection represents the strongest long-term prognostic factor and chance for cure. To remove microscopic or macroscopic RD, reoperation is recommended for T1b or higher IGBC by international guidelines (14). Consistent with previous studies, reresection is beneficial and associated with improved survival for patients with IGBC (4–6). Interestingly, resection margin status in our cohort did not show significant differences in prognosis for patients with IGBC after reoperation, which was also observed in the study of Vega and colleagues (20). However, the opposite conclusion can also be drawn from the work of de Savornin Lohman (4). The paradoxical results aroused our attention. Despite the improved survival observed in the reresection group, patients with RD have been shown to have shorter survival times than those without RD (4, 19). It is now evident that patients without residual disease or with disseminated disease cannot benefit from reoperation. Ramos’ group (18) showed that only the patients with local RD that isolated nondiscontinuous involvement of the vesicular bed or the cystic stump were found to have acquired more benefit from reoperation compared with regional or distant RD. Similarly, the conclusion that reresection may be beneficial solely for patients with microscopic RD undetected by the pathologist was made by the de Savornin Lohman group (4). They perceived that the tumor may have already progressed beyond potential curation when macroscopic RD was found. In this regard, we presume that the difference in predictive prognosis efficacy of resection margin status may be due to the varying proportion of patients who can potentially benefit from reresection. Consequently, the survival benefit of reoperation for T1b IGBC remains controversial (21, 22). The survival benefit of reoperation for T2/T3 IGBC patients has reached an expert consensus (6, 22).

Additionally, peritoneal metastasis occurred frequently in IGBC, mainly due to bile spillage of the gallbladder during initial cholecystectomy, particularly in minimally invasive approaches on various conditions. It was an important factor for IGBC patients in losing the chance of radical reoperation. Statistically, approximately 7-7.6% of patients with peritoneal metastasis were found to have reoperation (23, 24), which was similar to our results (6.3% of patients with peritoneal metastases during reoperation). Evidently, the poor prognosis association with peritoneal metastasis has also been demonstrated in multiple abdominal cancers, such as colorectal, gastric and liver cancers (25–27).

In addition, adenosquamous or squamous cell carcinoma represents a minority (2%) histological type of gallbladder cancer. Studies have shown that it is commonly larger and more aggressive than adenocarcinoma, with a significantly shorter median overall survival than adenocarcinoma, and is an independent prognostic factor for GBC (28, 29), which was similar to and supported our results.

In the present study, the proposed nomogram, which incorporated 5 comprehensive variables (including T stage, N metastasis, peritoneal metastasis, reresection and histology), performed well, as supported by the C index values of 0.76 and 0.814 in the training and validation cohorts, respectively, and the calibration curves showed excellent agreement between predicted and observed outcomes in the 1-, 3-, and 5-year OS. Remarkably, IGBC has unique characteristics, such as a few patients with distant metastasis and iatrogenic peritoneal metastasis often derived from bile spillage that occurred at initial surgery. Therefore, M status was excluded from the nomogram, while peritoneal metastasis and the other 4 variables were included in the nomogram, and the nomogram was more accurate than the AJCC TNM staging system for predicting the prognosis of patients with IGBC.

However, some limitations need to be considered in this study. First, it was a retrospective single-center study without external data validation, which may result in some bias and low accuracy, and further large-scale multicenter cohort studies are needed to validate our results. Second, the lack of relevant information on postoperative adjuvant chemotherapy and serum tumor markers may reduce the accuracy of our predictions, and future studies need to consider these variables. Despite these limitations, the nomogram model constructed in this study has excellent AUC values and calibration curves, making it an excellent model to provide physicians with accurate survival prediction.

## 5 Conclusion

In conclusion, based on the variables identified in this study, we successfully established a nomogram of IGBC for the first time. Well-calibrated nomogram survival curves can help physicians to make appropriate clinical decisions for individual IGBC patients.

## Data availability statement

The original contributions presented in the study are included in the article/supplementary material. Further inquiries can be directed to the corresponding authors.

## Ethics statement

This study was approved by the institutional Review Board of Shanghai Eastern Hepatobiliary Surgery Hospital Ethics Committee (No. EHBHKY2022-K-025) and complied with its ethical standards. The patients/participants provided their written informed consent to participate in this study. Written informed consent was obtained from the individual(s) for the publication of any potentially identifiable images or data included in this article.

## Author contributions

Acquisition of data, analysis and interpretation of data: Z-HX, XS, M-QL, JW, J-XZ, K-JC, WL, R-LG. Writing-original draft: Z-HX, XS, M-QL. Writing-review and editing: allauthors. Supervision: X-QJ, Q-BC. All authors contributed to thearticle and approved the submitted version.
